# Ternary V-Scheme Ag_2_WO_4_/BaO/NiO Heterojunction Photocatalysts: Very Fast Degradation Process for Congo Red under UV-Light Irradiation

**DOI:** 10.1021/acsomega.2c08090

**Published:** 2023-02-28

**Authors:** Ali İmran Vai̇zoğullar, Çağla Topkara, Mehmet Uğurlu

**Affiliations:** † Vocational School Health Care, Medical Laboratory Programme, Muğla Sıtkı Koçman University, Muğla 48000, Turkey; ‡ Faculty of Science, Department of Chemistry, Muğla Sıtkı Koçman University, Muğla 48000, Turkey

## Abstract

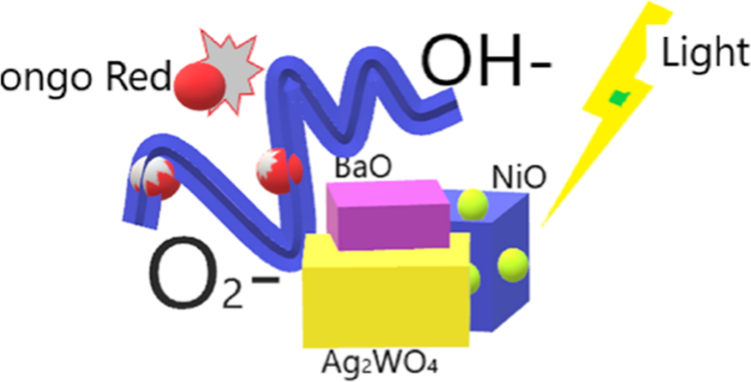

With increasing industrial production, pollutants generated in the process of bleaching or dying disperse to the natural water medium. Therefore, an effective photocatalytic material must be prepared for water treatment quickly. In the present study, a novel and effective V-scheme Ag_2_WO_4_/BaO/NiO heterostructure photocatalyst with high photocatalytic performance for the degradation of different organic pollutants was designed and formed by a simple precipitation method. Scanning electron microscopy images showed that BaO, NiO, and Ag_2_WO_4_/BaO/NiO have a nanopipe, spherical, and nanorod morphology, respectively. X-ray diffraction results indicated that cubic phases were obtained with higher crystallite structure and lower crystallite distortion. The optical properties of the samples exhibited UV-absorption regions with about 3.35, 3.38, and 3.28 eV band gaps for BaO, BaO/NiO, and Ag_2_WO_4_/BaO/NiO, respectively. The photocatalytic activity was investigated by the degradation of Congo red under UV-light irradiation. To investigate the photocatalytic mechanism, the photodegradation performance of the catalyst was analyzed with different scavengers such as isopropyl alcohol, ascorbic acid, and potassium iodide (KI), and it was shown that the main active species were ^•^O_2_^–^ radicals and that OH^•^ radicals have a significant contribution toward the degradation process. Compared to bare BaO and BaO/NiO samples, Ag_2_WO_4_/BaO/NiO showed excellent photocatalytic activity and about 41%, 66 and 99% of Congo red photodegraded under UV light within 30 min. The reason for this is that the Ag_2_WO_4_/BaO/NiO heterostructure displayed wider contact which promoted more charge-transfer ways to shorten the electron transportation path and increase the inhibition of electron–hole pairs.

## Introduction

Recently, with the expansion of industrialization development and effluent discharge, water pollution has significantly increased. To date, several methods have been used for water treatment such as photocatalytic degradation, adsorption, biological treatment, and flocculent precipitation. Among these processes, photocatalytic degradation has been the most effective technique for the removal of toxic or hazardous organic compounds due to the strong oxidizing potential of hydroxyl radicals (OH^•^) generated during the photocatalytic process.^1^ Especially, synthetic dyes are persistent organics due to their robust structure. As a result of chemical processes like oxidation and hydrolysis, these dyes discharge into the medium as hazardous byproducts.^2^ In addition, these azo dye compounds have stayed in the environments for a long time stably due to the complex aromatic structure. Moreover, the adverse effect such as serious allergies to humans, plants, and aquatic growth, even inducing cancerous cells causing tumors.^3^ In fact, it is difficult to remove these colored chemicals. Dyes are stable to biological and chemical treatment due to the transition of phases. This formation has attracted scientists to the effective decomposition of these toxic chemicals. In the recent decade, photocatalytic activation processes involving semiconductors have been used to clean wastewater. Moreover, it has become a method that plays an active role not only in photocatalytic degradation but also in many chemical processes such as bacterial inactivation and reduction reactions.^4^ In light of this explanation, it is necessary to develop a material that can be synthesized easily and used semiconductors with a regular morphology in order to eliminate water pollutants efficiently. Recently, photocatalysts such as NiO and SnO_2_ have been synthesized together with the most widely used TiO_2_. They also function as good sensors. However, materials containing BaO, NiO, and Ag_2_WO_4_ as ternary composites have started to be synthesized.^5^ BaO has often been used in hydrogen production. It has not been found to be used much in photocatalytic degradation studies. This result is due to the fact that the band gap energy of BaO is variable. The optical properties of BaO vary according to the synthesis method. For example, Hussain and co-workers (2019)^6^ have synthesized the BaO/TiO_2_ with a simple chemical reduction method where the band gap of BaO-based binary BaO/TiO_2_ has been obtained as 3.2 eV. On the other hand, Ansari and Jahan (2021)^7^ synthesized BaO particles with a chemical precipitation method with a 4.4 eV band gap value. Unlike BaO, NiO was synthesized as binary composites and used in photocatalytic degradation studies. For example, in a study by Gnanasekaran and co-workers, it was stated that the band gap energy decreases with the increase in the amount of NiO. The band gap energy of pure NiO was calculated as 3.62. This result shows that NiO and BaO give a more active absorption spectrum in the UV region.^3^ Ag-based semiconductors have gained the focus of researchers in the photocatalytic field due to their higher photocatalytic activity and wider light response range.^8^ Among them, Ag_2_WO_4_ is the most effective material with its unique properties such as high photocatalytic performance, good conductivity, and safety.^9^ In contrast, instability, aggregation tendency, and fast recombination of charge carriers show its negative aspects for the photocatalytic process. To overcome this, Ag_2_WO_4_ can be combined as a heterojunction, dopant, or cocatalyst structure.^10^

To date, there is no reported study of Ag_2_WO_4_/BaO/NiO photocatalysts for the degradation of some organic pollutants. After the combination with BaO/NiO, the electron transfer ways shortened, and effective inhibition was obtained in the Ag_2_WO_4_/BaO/NiO photocatalyst sample.

### Preparation of BaO, NiO, and Ag_2_WO_4_/BaO/NiO Photocatalyst Samples

Barium nitrate [Ba(NO_3_)_2_] and sodium bicarbonate (NaHCO_3_) were used as a precursor for the coprecipitation method. 1.36 g of Ba(NO_3_)_2_ was dissolved in 250 mL of deionized water (H_2_O) and stirred for 30 min until Ba(NO_3_)_2_ dissolved completely. 1.27 g of NaHCO_3_ was added to the solution under vigorous stirring. The obtained white solid was aged for 12 h at room temperature by covering. The precipitated sample was washed and centrifuged with water and ethanol. After that, it was dried at 80 °C and calcined at 450 °C for 2 h.

In order to synthesize the BaO/NiO sample, 0.12 g of BaO was dispersed in 250 mL of distilled water. 2.28 g of nickel nitrate hexahydrate [Ni(NO_3_)_2_·6H_2_O], 1.2 g of sodium hydroxide (NaOH), and 0.1 g of polyvinylpyrrolidone (PVP, MW = 65 000) were added to the solution. The mixed solution was stirred for 2 h at room temperature. The soot color precipitate was filtered and washed with distilled water and ethanol. Then, it was calcined at 450 °C for 2 h.

To prepare the Ag_2_WO_4_/BaO/NiO sample, 1.1 g of the BaO/NiO sample was dispersed in 250 mL of distilled water. To this, 0.4 g of AgNO_3_ and 0.1 g of PVP were added with 25 mL of deionized water (solution A). 1.2 g of Na_2_WO_4_, 0.1 g of PVP, and 30 mL of deionized water were stirred in a different beaker (solution B). Solution B was added to solution A drop by drop to form a ternary sample. The obtained mixed precipitate in shades of yellow, black, and white color was dried at 90 °C for 2 h and calcined at 450 °C for 3 h. In the Scavenger experiments, to reveal major active species such as OH^•^, ^•^O^2–^, and h^+^, 1 mM isopropyl alcohol, ascorbic acid, and potassium iodide (KI) were used, respectively.

### Characterization of the Photocatalyst Samples

The crystalline structure of the samples was examined by X-ray diffraction (XRD): Rigaku D/MAX 350) using copper K radiation k = 0.154056 nm). The morphological structure and EDS mapping analyses of the particles were performed using [scanning electron microscopy(SEM)] JEOL JSM7600F). The photoluminescence (PL) emission spectra of the samples were obtained using a spectrofluorometer (Spex 500 M, USA). Raman studies were performed at room temperature using a Raman spectrophotometer (Bruker IFS 66/S, FRA 106/S, HYPERION 1000, RAMANSCOPE II). The recorded spectra were obtained using 532 nm lasers. X-ray photoelectron spectroscopic (XPS) measurement was performed using a PHI 5000 Versa Probe. The electrochemical impedance spectra were analyzed on an impedance analyzer (Gamry Potansiyostat/Galvanostat/ZRA Reference 3000) using a standard three-electrode system with the samples as the working electrodes, a saturated calomel electrode as the reference electrode, and a Pt wire as the counter electrode. The frequency operating range was specified as 1 kHz to 107 Hz. The UV–vis DRS analyses of all the samples were performed using a Lambda 35 UV–vis spectrophotometer in the solid state.

## Results and Discussion

### SEM Analyses

**Figure 1:**
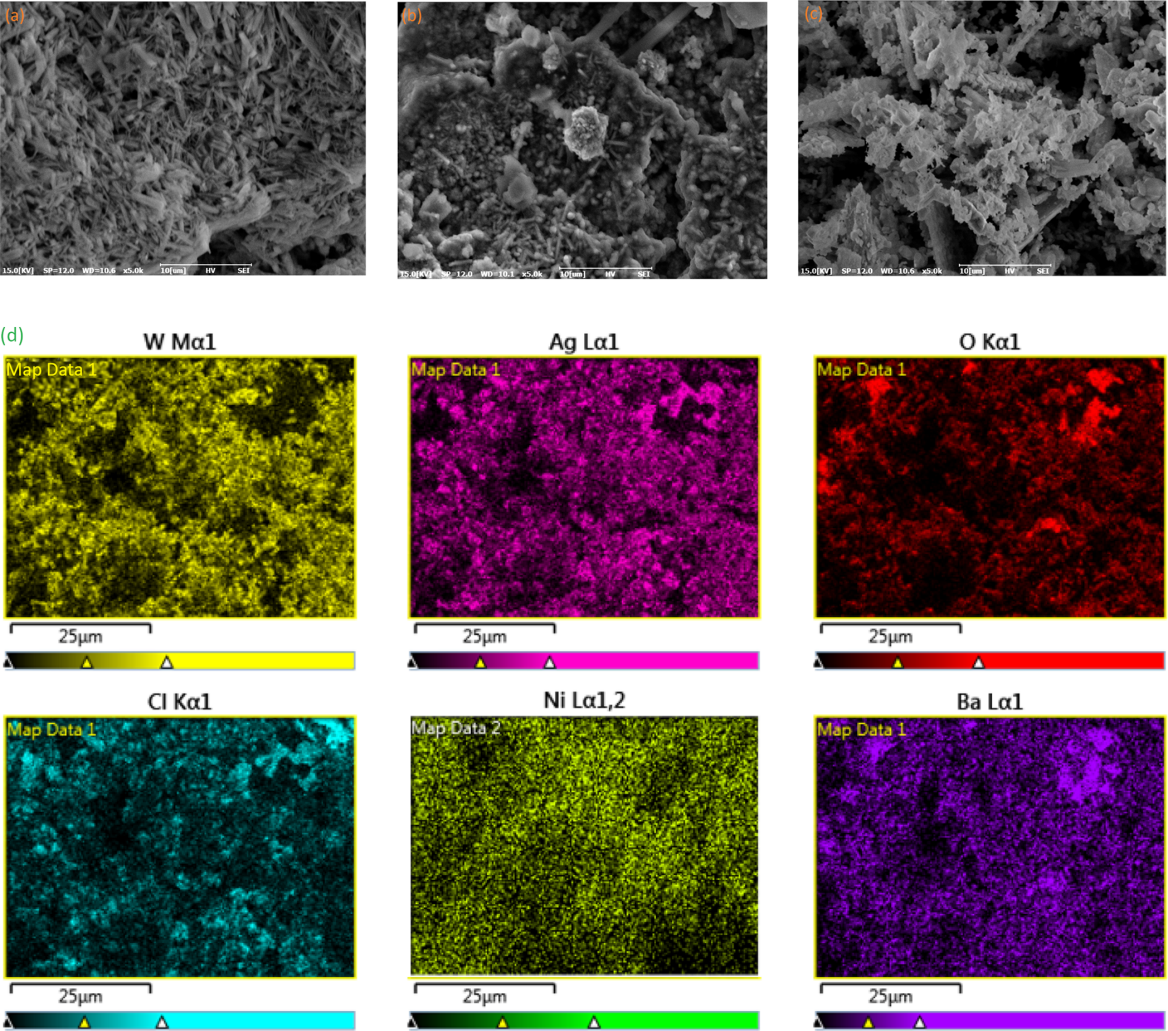
SEM images of BaO (a), BaO/NiO (b), and Ag_2_WO_4_/BaO/NiO (c). Elemental Mapping results (d).

The morphology of as-prepared photocatalysts samples was evaluated by SEM. Figure 1 presents the SEM images of samples. It was observed that bare BaO shows a nanopipe morphology with a 400–500 nm thickness approximately (Figure 1a). All particles have almost the same length and lower aggregation. While it was synthesized in the composite form with NiO (NiO), there was not much change in the morphology of BaO itself. In addition, spherical NiO was effectively dispersed on BaO nanopipes. The resulting NiO formed as a nanosphere morphology. However, in such a case, a slight increase in the thickness and length of BaO was observed (Figure 1c). A possible explanation for this might be van der Waals and electrostatic forces. These two forces are in opposition to each other. Electrostatic forces repel, and van der Waals forces attract.^11^ The tendency to have a lower surface area made the NiO particles exert pressure on the BaO particles. The electrostatic forces maximized this pressure, resulting in interparticle repulsions in particle formation and causing an increase in size. In Figure 1c, it is clearly seen that three different morphological structures formed. Both NiO nanospheres and BaO nanopipes were aggregated on Ag_2_WO_4_ nanorods. This formation caused a bush-like shape of Ag_2_WO_4_ particles. These findings suggest an effective heterojunction photocatalyst sample for having higher photocatalytic performance in both degradation and H_2_ production reactions.

EDX elemental mapping was performed to obtain quantitative results of the prepared materials (Figure 1d). As seen, Ag, Ba, Ni, W, and O elements are present in the most photoactive Ag_2_WO_4_/BaO/NiO sample with some impurities such as chlorine. The space distribution of the elements in the Ag_2_WO_4_/BaO/NiO was obtained by EDX. The results display the locations of different elements in the selected area with a well-defined spatial distribution. The mass percentages of Ag, W, Ba, and Ni were estimated as 4.41 Ni, 8.55 Cl, 41.84 Ag, 5.43 Ba, 29.71 W, and 7.76 O, respectively. These results were slightly different from the theoretical values.

### XRD Analyses

**Figure 2:**
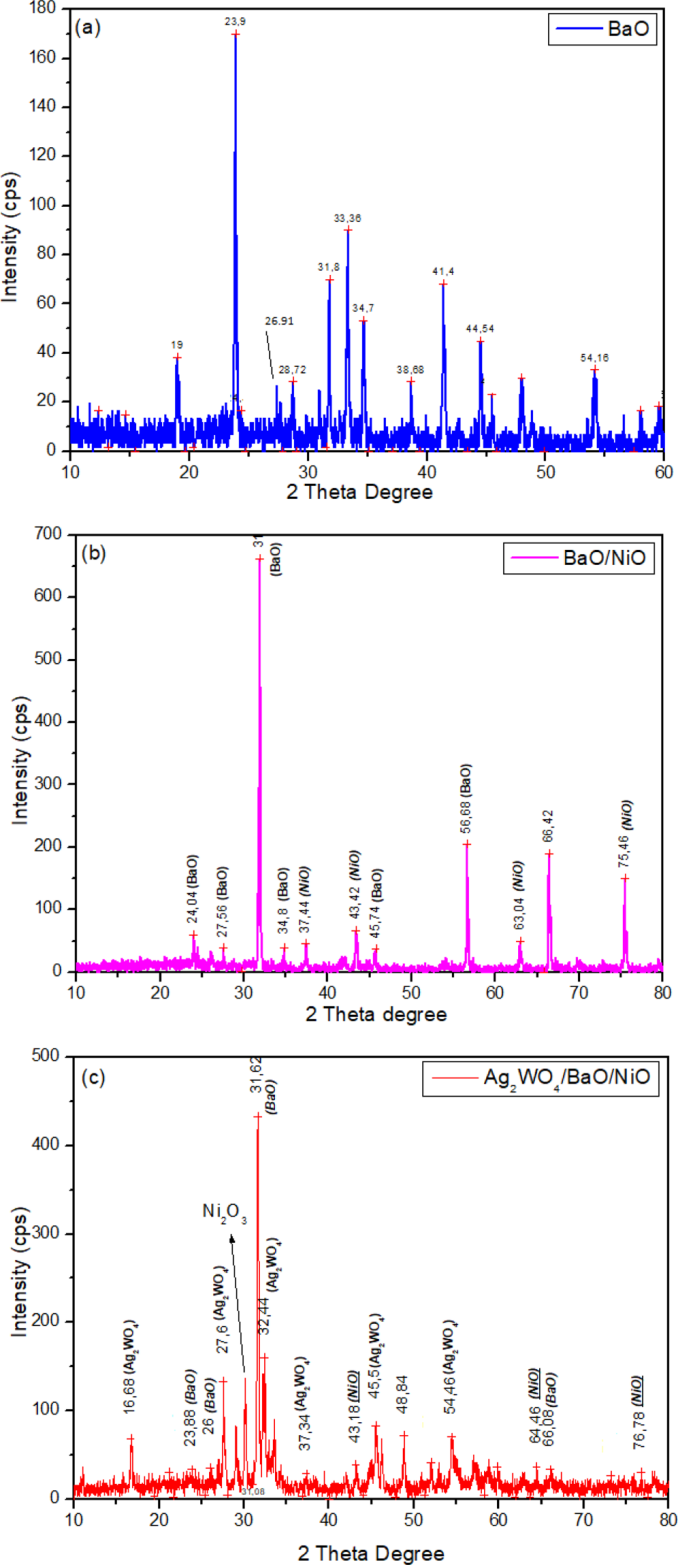
XRD patterns of BaO (a), BaO/NiO (b), and Ag_2_WO_4_/BaO/NiO (c).

Figure 2 presents the XRD patterns of the samples. Bare BaO (Figure 2a) shows 19.0, 26.91, 33.3, 44.5, and 54.1° degrees attributed to the (1 2 0), (1 0 1), (1 1 0), (1 0 3), and (2 1 1) planes, respectively, indicating the body-centered structure.^12^ In addition, other peaks at 23.9, 28.72, 31.8, 34.7, 38.6, and 66.44° degrees were attributed to the (2 0 1), (1 0 2), (2 0 0), (2 1 2), (1 1 1), (2 0 0), and (4 0 0) planes, respectively, confirming the tetragonal phase of BaO nanoparticles.^7^ It is understood from these results that BaO synthesized by a simple chemical precipitation method can be obtained in different phases. In the composite form of BaO with NiO (BaO/NiO), the characteristic NiO peaks were seen at 37.4, 43.4, 63.0, and 75.4° degrees, which corresponded to the (1 1 1), (2 0 0), (2 2 0), and (3 1 1) planes, respectively. It is clearly shown that all diffraction peaks can be indexed to the face-centered cubic crystalline structure of NiO. Also, the relative intensity of the peaks and peak position overlapped with that of the standard spectrum (JCPDS, no. 04-0835).^13^ Other diffraction peaks at 24.0, 27.5, 31.9, 34.8, 45, 74, 56, 68, and 66.42° degrees were attributed to the BaO in the BaO/NiO sample. NiO also presents a single phase and no other impurity matter. As seen from Figure 2a,b, the most intense peak of BaO at 23.9° decreased in Figure 2b. A possible explanation for this may be that NiO particles were well dispersed on the (2 0 1) plane. Also, the most intense peak of BaO at 31.9° in Figure 2b decreased. This indicates that the orientation and growth in the size of the crystal occurred in the (2 0 0) plane. The ternary Ag_2_WO_4_/BaO/NiO composite presents Ag_2_WO_4_ diffraction peaks with BaO and NiO together. The diffraction peaks at 16.6, 27.6, 32.4, 37.8, 45.1, and 54.6° degrees of the (1 1 0), (0 1 1), (4 0 0), (2 4 1), (4 0 2), and (2 3 3) planes, respectively, were attributed to the cubic phase of Ag_2_WO_4_. Surprisingly, the Ni_2_O_3_ main peak at 31.08° shows that the second phase of NiO was observed.^14^ A possible explanation for this may be that a small amount of Ag^1+^ ions can be reduced, while Ni^2+^ ions can be oxidized. The small variations in diffraction peaks are related to the deformations and size of the bonds.^15^ The lattice parameters of Ag_2_WO_4_, BaO, and NiO are shown in Tables 1 and 2. As can be seen from Tables 1 and 2, the lattice parameters of BaO and NiO in the BaO/NiO sample increased while decreasing in the Ag_2_WO_4_/BaO/NiO sample due to Ag_2_WO_4_. This result shows that the components are incorporated in each other to a small extent, which reduces the crystallite distortions.^16^

**Table 1:** Lattice Parameters of BaO, NiO, and Ag_2_WO_4_

	lattice parameters (Å)			
samples	*a*	*b*	*c*	refs
BaO	*4.76*	*4.76*	*3.95*	(17)
NiO	*4.22*	*4.22*	*4.22*	(18)
Ag_2_WO_4_	*9.33*	*9.33*	*9.33*	(19)

**Table 2:** Calculated Lattice Parameters of BaO, NiO, and Ag_2_WO_4_ in Composite Samples and Structural Parameters of BaO, BaO/NiO, and Ag_2_WO_4_/BaO/NiO Samples

	lattice parameters (Å)		lattice parameters (Å)							
samples	BaO	NiO	BaO	NiO	Ag_2_WO_4_	dislocation density (δ)	microstrain (∈)	stacking fault (SF)	kinetic rate constant (k^–1^)	*R*^2^
BaO sample						*0.0144*	*0.0366*	*0.105*	0.016	*0.97*
BaO/NiO sample	*a* = *4.77b* = *4.77c* = *3.97*	*a* = *4.23b* = *4.23c* = *4.23*				*0.0318*	*0.0340*	*0.088*	0.033	*0.98*
Ag_2_WO_4_/BaO/NiO sample			*a* = *4.75b* = *4.75c* = *3.92*	*a* = *4.24b* = *4.24c* = *4.24*	*a* = *9.36b* = *9.36c* = *9.46*	*0.0382*	*0.0470*	*0.122*	0.067	*0.98*

The observed sharp and intense peaks in Figure 2a–c display the synthesized nanoparticles that are highly crystalline in nature. The average crystallite size (*D*) of the sample is calculated using Debye–Scherrer’s formula which can be given as *D* = *K*λ/β Cos θ, where *K* is the wavelength of the CuK_α_ radiation [1.54060 Å], β is the full width at half-maximum of the diffraction peak, and θ is the angle of diffraction. The most intense peak was used to calculate the crystallite size. The obtained average sizes of all photocatalysts samples were 8.33, 5.60, and 5.11 nm, respectively. This result also coincides with the change in lattice parameters.

Also, the dislocation density, microstrain, and stacking default for all samples were calculated using the following equations:
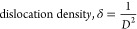

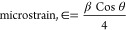

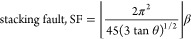


According to these equations, the calculated values are shown in Table 2. As seen from Table 2, the microstrain is found to be positive for all samples, indicating the influence of tensile forces on crystal structures.^20^ In addition, higher values of microstrain and dislocation density of the Ag_2_WO_4_/BaO/NiO sample present the existence of more lattice imperfections and structural defects in the Ag_2_WO_4_/BaO/NiO nanostructure.^21^ Also, different stacking modes can change the threshold energy of intermediates and the transfer of charge carriers to modulate the photocatalytic activity.^22^ These findings also confirm that an efficient photocatalyst can significantly degrade organic pollutants very fast under UV-light irradiation.

### XPS Analyses

**Figure 3:**
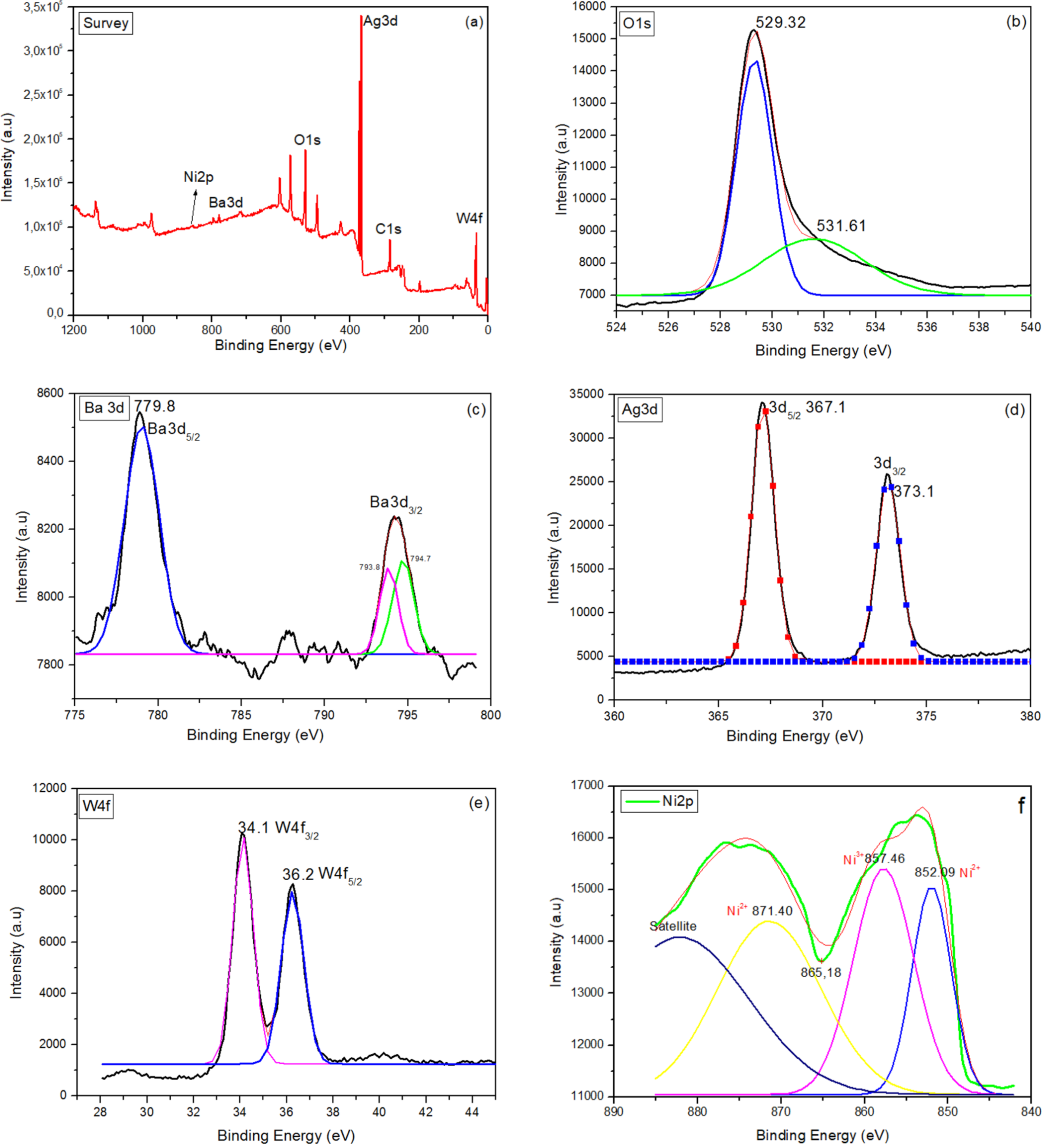
XPS analyses of survey (a), oxygen (b), barium (c), silver (d), tungsten (e), and nickel (f).

XPS measurements were conducted to explain the chemical states of the elements in the Ag_2_WO_4_/BaO/NiO sample. Figure 3a shows the survey spectra of the Ag_2_WO_4_/BaO/NiO composite, indicating the coexistence of Ag, W, O, Ba, and Ni. The peak centered at 529.32 and 531.61 eV, assigned to O^2–^ and chemically adsorbed oxygen (O^chem^) on the surface, respectively.^23^ The Ba 3d peaks for the Ag_2_WO_4_/BaO/NiO sample are composed of XPS lines corresponding to Ba3d_3/2_ and Ba3d_5/2_ spin–orbit coupling pairs for Ba3d_5/2_ positioned at higher and lower binding energies, respectively.^24^ The deconvolution using Gaussian showed that higher binding energy has two visible components. The fitting has two components at 793.8 and 794.7 eV for Ba3d_3/2_ showed that Ba atoms in the Ag_2_WO_4_/BaO/NiO sample oxides form relaxed Ag_2_WO_4_/BaO/NiO phases due to the residual defects and oxygen vacancy.^25,26^ In Figure 3d, the Ag3d peaks at 367.1 and 373.1 eV are two doublets fitted for silver. The peak at 373.1 eV shows 3d_3/2_ of Ag^1+^, while the peak at 367.1 confirms the 3d^5/2^ of the Ag^1+^ oxidation state.^27^Figure 3e shows that the W 4f spectra consist of spin–orbit split doublet peaks attributed to the W 4f_7/2_ and W 4f_5/2_ states. These two peaks suggest the W^6+^ state. In the Ni 2p spectra, three peaks can be observed. The peaks of Ni^2+^ at 852.04 eV and 871.40 eV were attributed to the 2p_3/2_ at and 2p_1/2_, respectively. However, the peak at 857.46 eV was of 2p_3/2_ of Ni^3+^ in Ni_2_O_3_.^28^ When all XPS analyses are examined, the oxidation steps overlap compared to previous studies, but the spectra show some shift to the right or left. This result suggests that there are large oxygen vacancies in the composite or some deterioration of the crystal.

### UV-DRS Evaluation

**Figure 4:**
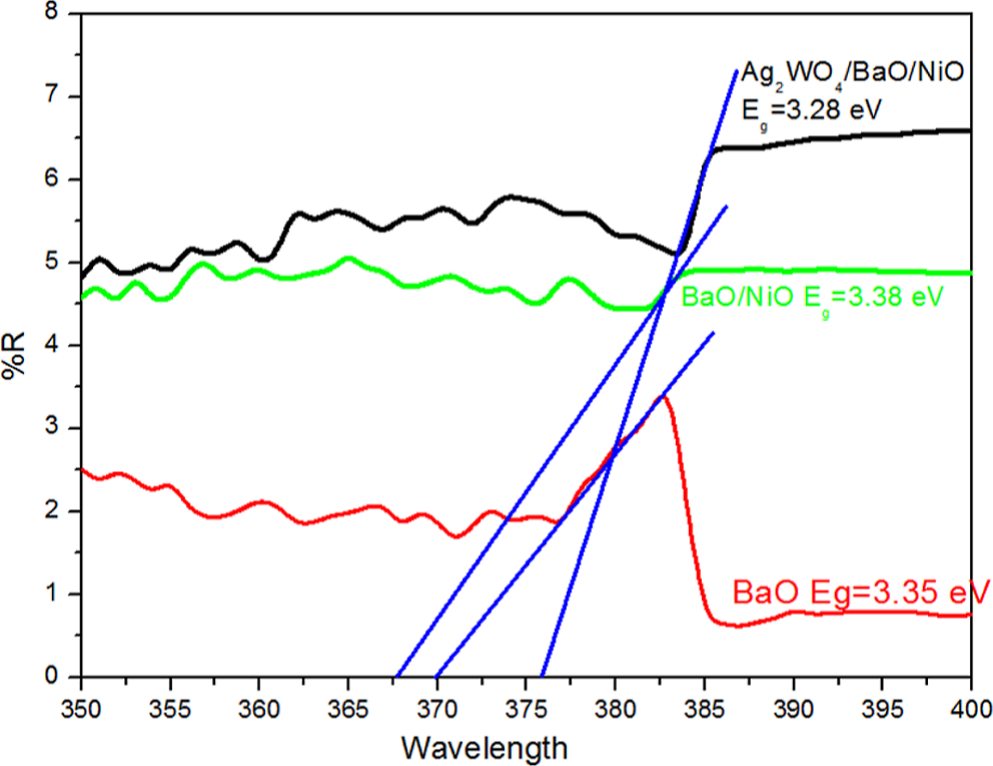
UV-DRS spectra of the samples.

The light absorption properties play an important role in explaining the photocatalytic performance of semiconductor materials. The corresponding band gap can be calculated. Figure 4 presents the UV-DRS results of photocatalyst samples. In Figure 4, the wavelength ranges between 350 and 400 nm. Meanwhile, the optical absorption for the BaO, BaO/NiO, and Ag_2_WO_4_/BaO/NiO samples is seen at about 370, 366, and 378 nm, respectively. This indicates that the primitive BaO, NiO, and Ag_2_WO_4_ are effective in the UV-light region. Interestingly, the band gap energies of the composites decrease even in the UV region. However, when the previous studies were examined, it was observed that the band gap energy of pure BaO, NiO, and Ag_2_WO_4_ was lower than the values obtained in these studies. A possible explanation for this may be the quantum size effect. The size of the nanoparticle is smaller than the de Broglie wavelength. In this case, the electrons and holes are spatially confined and discrete electronic energy levels would be formed in all materials. The band gap energy of the samples was calculated by the following equation
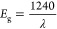
where λ is the wavelength of the *x*-axis. The *E*_g_ values of BaO, NiO/BaO, and Ag_2_WO_4_/BaO/NiO are shown in Figure 4. It can be seen from Figure 4 that the Ag_2_WO_4_/BaO/NiO sample has a smaller band gap value than that of another. The optical red shift of the band edge of the Ag_2_WO_4_/BaO/NiO sample can be attributed to the effect on the heterostructure, crystalline, and surface composition. The calculation of the conduction band and valence band of BaO, NiO, and Ag_2_WO_4_ samples was done using the following formula.
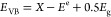

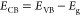
where *E*_VB_ and *E*_CB_ are the valence and conduction band edge potential, respectively, and *X* is the absolute electronegativity of the semiconductor. *E*^e^ is the energy of free electrons on the hydrogen scale with 4.5 eV. Using Pearson’s absolute electronegativity, the absolute electronegativity of Ba, O, Ni, Ag, and W is 2.40, 7.54, 4.40, 4.44, and 4.40, respectively. According to this, , , and .

**Scheme 1:**
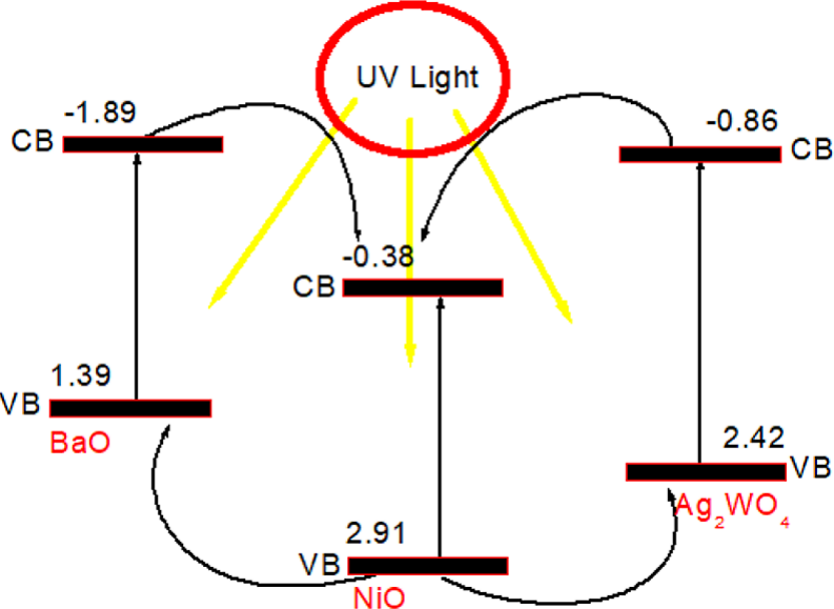
Possible Degradation Pathway of the Composite Ag_2_WO_4_/BaO/NiO

The calculated band edge potentials of the bare BaO, NiO, and Ag_2_WO_4_ are shown in Scheme 1. From Scheme 1, the CB edge potential of BaO is more electropositive and NiO is more electronegative. Differently, the VB potential of NiO is more electronegative than that of BaO and Ag_2_WO_4_. This result formed a “V” shape ternary heterojunction photocatalyst in the UV active. A semiconductor material is excited by UV or visible light; the electrons can jump from the VB level to the CB level to form excitons. However, it is difficult to separate completely due to the electrostatic attraction. To separate completely, free photogenerated electron and hole pairs are necessary by the external electric field force. This built-in electric field created by the conductive material plays an active role in the separation of electrons and holes. When the strength of this built-in electric field is not enough, the charge carrier concentration will start to increase out of equilibrium in the energy band of the material. This will cause the excited electrons to recombine. In order to suppress this, there must be an interface connection between the semiconductors. This link optimizes the migration paths of induced electrons and holes.^29^

### PL Analyses

**Figure 5:**
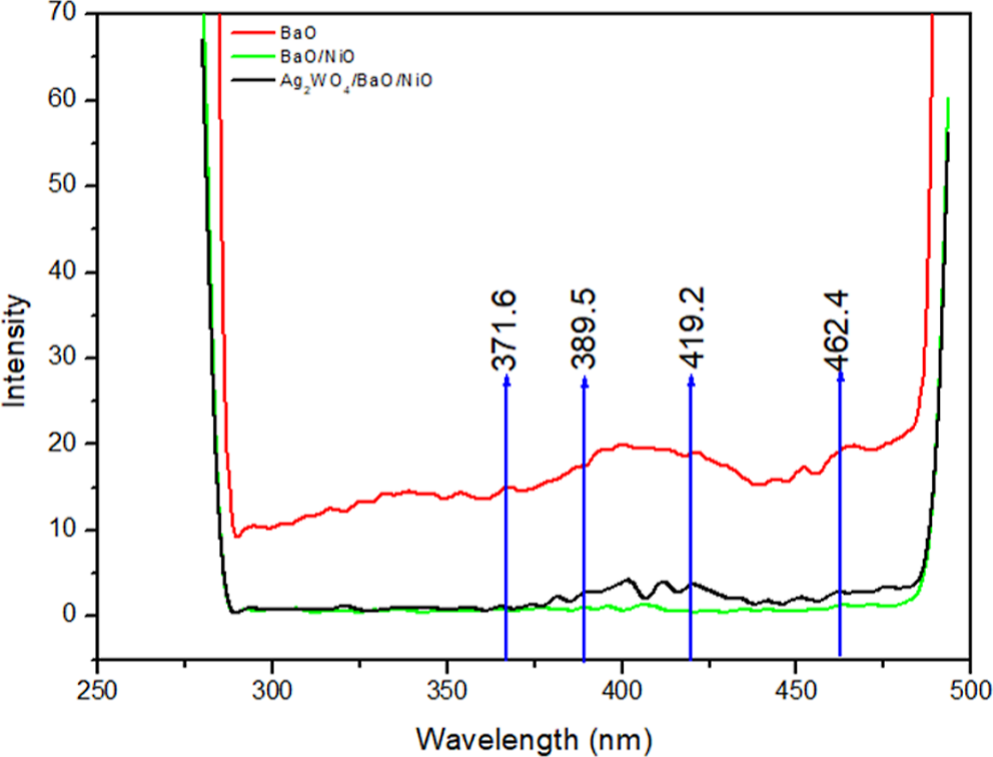
PL spectra of the samples (260 nm exciton).

Heterojunction composites can significantly inhibit the electron–hole pairs, which are confirmed by PL spectra. As known, the lower PL intensity presents the higher electron–hole transfer and separation efficiency of photogenerated species. It can be seen from Figure 5 that the samples are composed of several slight peaks located in UV and visible regions. In the visible region, 419.2 and 462.4 nm may be attributed to the defect-related emission peaks confirmed because of the intrinsic defects and intensely trapped oxygen vacancies. On the other hand, in the UV region, two emission peaks at 371.6 and 389.5 nm can be related to the recombination of electron–hole pairs.^30^ Also, as can be seen from Figure 5, pure BaO shows the strongest PL intensity, indicating higher recombination. Another composite BaO/NiO exhibits a lower intensity than pure BaO, suggesting that the loading of NiO supports the charge separation. Surprisingly, BaO/NiO and Ag_2_WO_4_/BaO/NiO composites show PL intensity close to each other. In addition, lower PL intensity of Ag_2_WO_4_/BaO/NiO is slightly higher than that of BaO/NiO indicating that some crystallite distortion can be formed in the ternary structure.

### Raman Analyses

**Figure 6:**
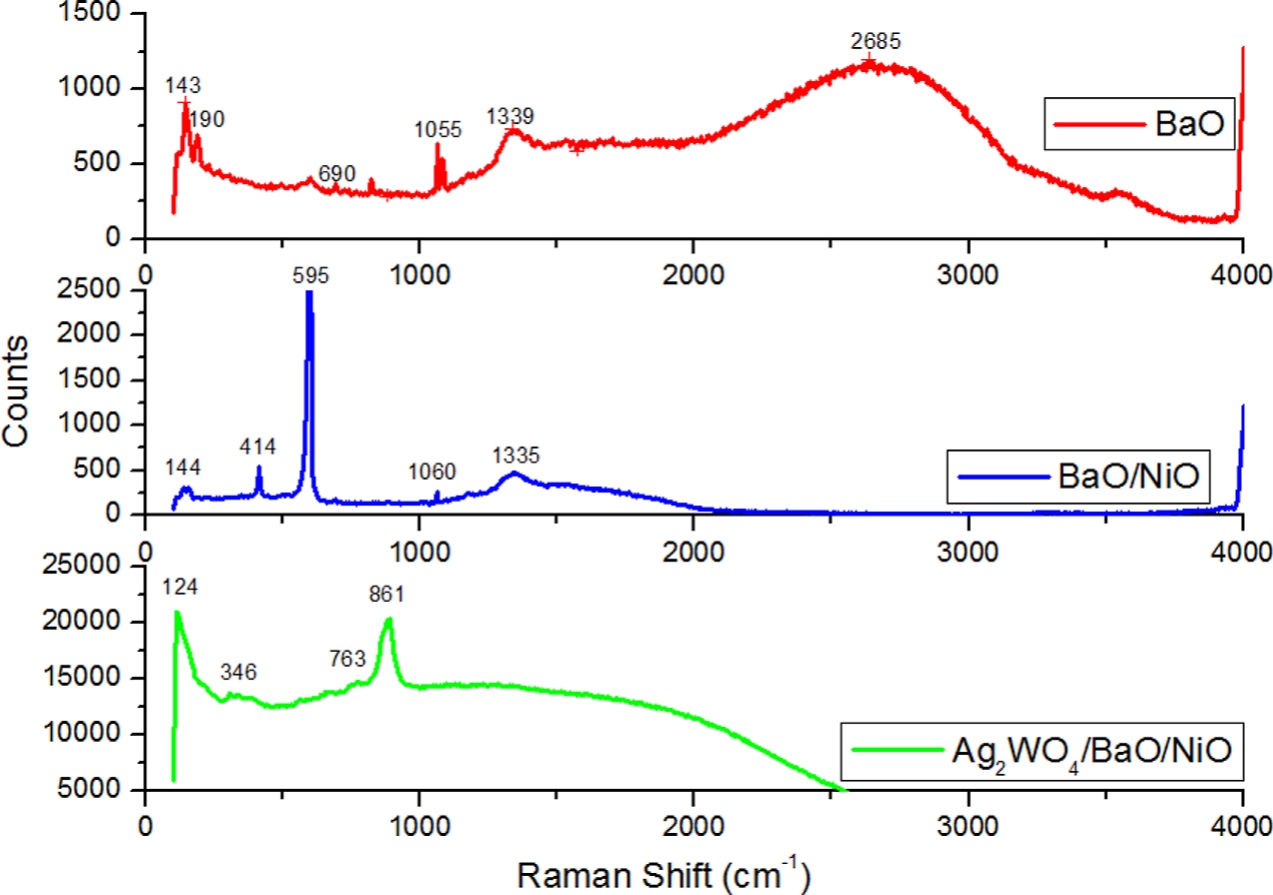
Raman spectra of the samples.

The Raman spectrum of the samples at room temperature and atmospheric pressure is shown in Figure 6. The Raman spectra of bare BaO present six phonon peaks at 143, 190, 690, 1055, 1339, and 2685 cm^–1^. Among them, the peak at 2685 cm^–1^ is due to the BaCO_3_ after CO_2_ adsorption by BaO under a standard atmosphere. The peak seen at 1055 cm^–1^ is attributed to the O–O stretching modes of vibration. The band at 690 cm^–1^ is connected to the Ba–O bond formation.^31^ The Raman spectrum of BaO/NiO shows dominant NiO scattering with one-phonon 414 cm^–1^ (TO) and 595 cm^–1^ (LO) modes. When Ag_2_WO_4_/BaO/NiO was evaluated, the Raman modes below 250 cm^–1^ are attributed to the translational lattice vibrations of Ag^+^ and W^6+^, mainly contributed by the motion of heavy Ag^+^ ions. The mode at 346 cm^–1^ is due to the bending vibrations of O–W–O, WOOW, and W–O–W. The mode at 763 and 861 cm^–1^ is the asymmetric stretching modes of W–O–W and W–O and the symmetric stretching of W–O, respectively.^32,33^ The modes of BaO and NiO are not fully observed in Ag_2_WO_4_/BaO/NiO. It is likely that the enlarged peak after 860 cm^–1^ covers BaO and NiO modes.

### Electrochemical Impedance Studies

**Figure 7:**
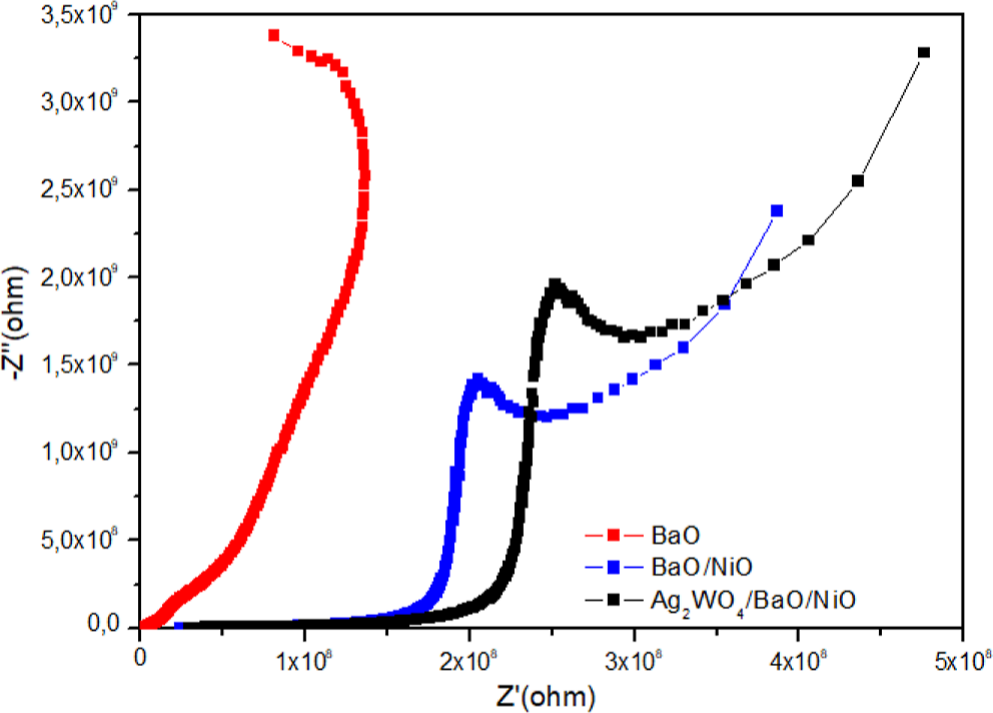
EIS of the samples (Nyquist plots).

To explore the electron transfer kinetics and interface properties, all samples were analyzed by EIS measurements. Figure 7 presents the Nyquist plot of each sample. As known, the smaller radius of the semicircle in the Nyquist plot means the electron can transfer more rapidly between the electrode and electrolyte. The impedance of BaO, BaO/NiO, and Ag_2_WO_4_/BaO/NiO decreases in turn, indicating the higher charge separation and transfer of Ag_2_WO_4_/BaO/NiO.^34^ Additionally, a decrease in the slope of the obtained line and the shift of the curve toward the Ź (ohm) axis in the Ag_2_WO4/BaO/NiO sample can be observed, which also shows that the conductivity in the sample is higher than that of BaO and BaO/NiO samples.^31^

### Photocatalytic Activity and Scavenger Studies

The photocatalytic performance of the samples was investigated using Congo red model pollutants. The maximum absorption of Congo red (10 ppm) was noticed at 496 nm in a UV–vis spectrophotometer. The degradation performances of the samples were monitored using this UV spectrophotometer. The UV–vis intensity of Congo red decreased as time increased. Also, a color change was seen. Congo red contains an azo (−N=N−) chromophore and an acidic auxochrome (−SO_3_H) associated with the benzene structure. Congo red is also called acidic diazo dye.^30^ Color changes are observed as a result of the degradation of these chromophore groups. The degradation efficiency of the samples was calculated using the following formula
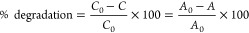
where C and A are the concentration and the absorbance, respectively. Since the concentration and absorbance are directly proportional to each other, therefore, the change in absorbance was accepted as a direct decrease in concentration. In order to explain the efficiency and feasibility of Congo red degradation, a kinetic study was performed. According to the Langmuir–Hinshelwood model below
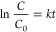
where *C*_0_ and *C* are the concentration of Congo red. The rate constant value is calculated on the graph of the concentration plotted against time. The closer the correlation coefficient to 1, the higher the linearity obtained. As known, in a photocatalytic reaction, there are two parameters to enhance a photocatalyst, which are higher separation and quick migration of charge carriers.^35^

**Figure 8:**
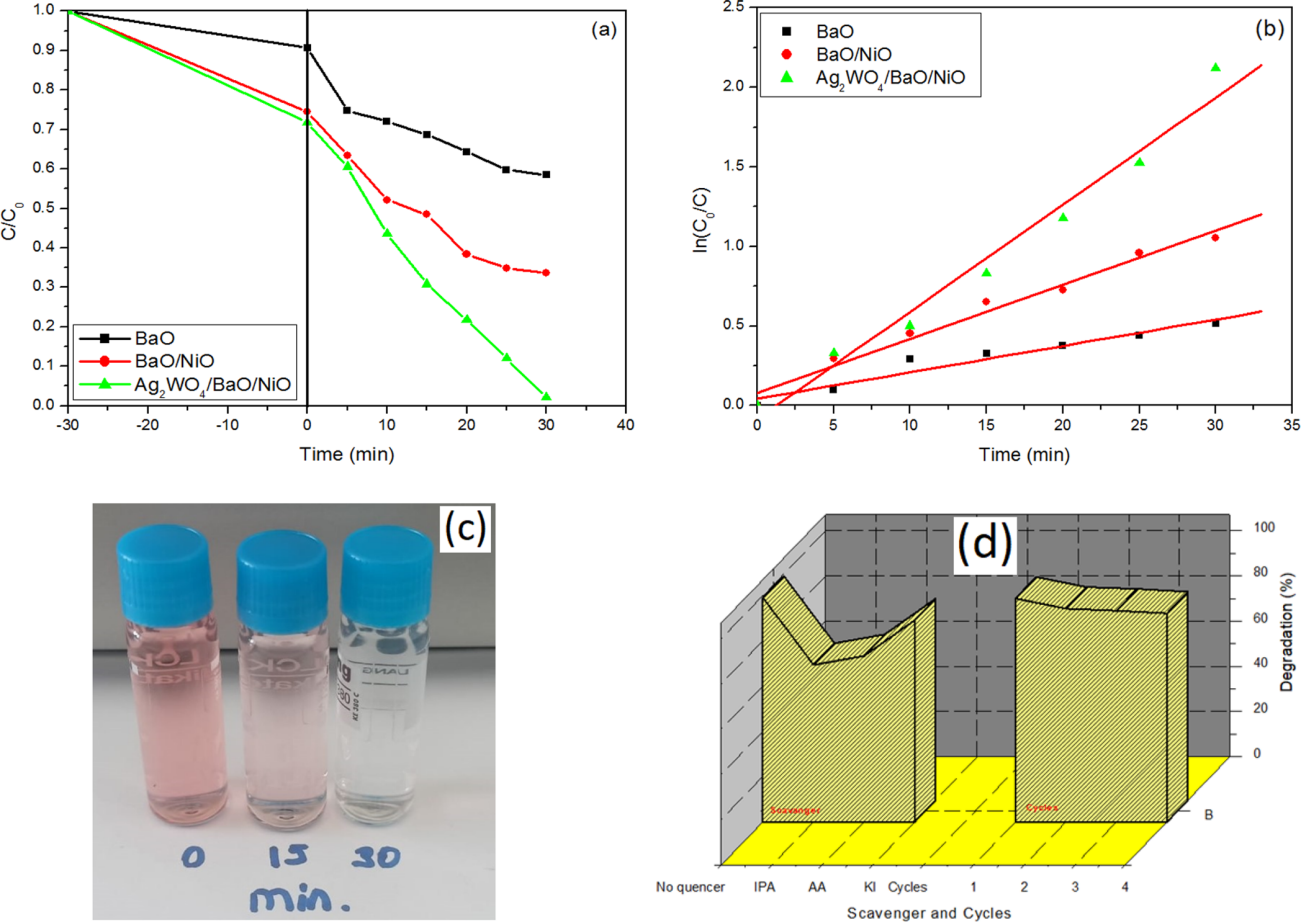
Photocatalytic degradation results of the samples (a), pseudo-first-order kinetic model of the samples (b), and decolorization results after 30 min degradation ***(This photo was prepared by authors*** (c). Scavenger and cycling results (d).

The photocatalytic performance of the samples is shown in Figure 8. Since the data obtained in the photolysis carried out in the catalyst-free medium are too small to be considered, it is not shown in Figure 8. However, some reduction was observed with mixing in the dark. This result shows that Congo red is adsorbed to the catalyst surface. About 41, 66, and 99% of Congo red photodegraded under UV light within 30 min (Figure 8a). These results were attributed to the inhibition of charge carriers effectively. On the other hand, BaO showed a lower degradation percentage. The degradation efficiency of BaO/NiO increased when BaO was composited with NiO. This result suggests that NiO enhanced the surface properties, stability, and reduction of aggregation.^36^ The kinetic rate constant of the samples is shown in Table 1. From this table, the correlation coefficients were closer to 1, indicating the pseudo-first-order kinetic model for the degradation of Congo red with BaO, BaO/NiO, and Ag_2_WO_4_/BaO/NiO (Figure 8b). Also, the kinetic rate constant of Ag_2_WO_4_/BaO/NiO was four and two times higher than that of BaO and BaO/NiO, respectively. This indicates that the Ag_2_WO_4_/BaO/NiO sample is more efficient and degrades Congo red under UV light fast. Based on the above explanation, more excellent information for the effective photocatalytic mechanism of ternary Ag_2_WO_4_/BaO/NiO heterojunction photocatalysts can be proposed. The calculated CB and VB edge potentials of BaO, NiO, and Ag_2_WO_4_ are −1.89, −0.38, and −0.86 eV and 1.39, 2.91, and 2.42 eV, respectively. Under UV-light irradiation, all three components can be excited. The photogenerated electrons from the *E*_CB_*of* BaO and Ag_2_WO_4_ transfer to the *E*_CB_ of the NiO. Simultaneously, holes from the *E*_VB_ of NiO can transport to the *E*_VB_ level of BaO and Ag_2_WO_4_. These processes of charge carriers highly increased the recombination inhibition of electron/hole pairs. The presence of NiO can act as a “V” scheme transfer pathway. Radical trapping results showed that OH^•^ radicals contributed to the photocatalytic mechanism with the following reaction.^37^
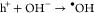
When Scheme 1 is re-examined, it can be clearly seen that more electronegative NiO attracts the excited electrons to its CB from the CBs of BaO and Ag_2_WO_4_. Similarly, holes in the VB of NiO can transfer to the VBs of BaO and Ag_2_WO_4_. The distribution of different electrons and cavities of this ternary composite prevents the recombination of electron and hole pairs, making it an effective photocatalytic material under UV.

The possible charge transfer pathway can be explained as below. The transferred electrons from CB level of BaO and Ag_2_WO_4_ to the NiO CB level, due to the more negative standard redox potential of O_2_/^•^O_2_ (−0.33 V), the accumulated electrons in the CB level of NiO can produce a superoxide radical to degrade Congo red. On the other hand, the VB potential of the components except Ag_2_WO_4_ was much more positive than the standard redox potential of OH^–^/OH^•^ (+1.99 V) and H_2_O/OH^•^ = +2.34 V,^38^ indicating that the (OH^•^) radicals from both OH^–^ and H_2_O can directly oxidize Congo red. In order to explain the reaction mechanism in detail, the main active species are defined in free radical trapping experiments investigated during the reaction process. In the present study, to reveal major active species such as ^•^OH, ^•^O_2_^–^, and h^+^, 1 mM isopropyl alcohol, ascorbic acid, and KI were used, respectively. The experimental results are shown in Figure 8d. As seen, the addition of isopropyl alcohol and ascorbic acid caused clearly lower degradation efficiency of the Ag_2_WO_4_/BaO/NiO sample. The removal percentage was obtained as 73.5 and 69.4% with isopropyl alcohol and ascorbic acid, respectively, demonstrating the significant role of the two radicals. On the contrary, 1 mM KI showed little effect on the degradation of Congo red, indicating that holes showed a weak performance in the reaction. The obtained results consisted of the scavenger experiments, showing that the main active species were ^•^O_2_^–^ radicals and OH^•^ radicals.

### Recyclability and Stability

The recycling experiments of Ag_2_WO_4_/BaO/NiO for the photocatalytic reaction under UV-light irradiation were performed to explain the optical stability of the catalyst. As seen in Figure 8d, about 93.1% is degraded after four runs, indicating that Ag_2_WO_4_/BaO/NiO has a little loss of photocatalytic activity. Also, during oxidation, the photocatalytic performances of Ag_2_WO_4_/BaO/NiO were stable, indicating lower photocorrosion.^39^

## Conclusions

We present for the first time the preparation and degradation performances of BaO, BaO/NiO, and Ag_2_WO_4_/BaO/NiO photocatalysts. The structural morphology of the samples was the cubic phase of the components. Also, they exhibited lower wavelengths in optical properties. The XRD studies displayed the development of cubic structures of the composite catalysts. The prepared samples were analyzed with SEM and showed nanopipes, spherical, and nanorod morphologies. Although some agglomeration was observed on the materials, no decrease in agglomeration-induced efficiency in photocatalytic degradation was observed. When the results of the XRD analysis were examined, it was determined that there were some deteriorations in the crystal structures of the materials. In addition, scavenger studies showed that O_2_^–^ and OH^•^ radicals play an active role in the degradation process. This confirmed that the very fast degradation time is due to the crystal structure of the material, and an effective electron–hole inhibition is achieved.
